# Antimicrobial and quorum sensing interference of a cysteine- and arginine-deleted linear Tachyplesin analog (CRDT) against *Staphylococcus aureus* and *Pseudomonas aeruginosa*

**DOI:** 10.1371/journal.pone.0334547

**Published:** 2025-10-10

**Authors:** Sirijan Santajit, Techit Thavorasak, Thida Kong-Ngoen, Nawannaporn Saelim, Thapani Srisai, Pisinee Aiumurai, Wanpen Chaicumpa, Nitaya Indrawattana

**Affiliations:** 1 Department of Medical Technology, School of Allied Health Sciences, Walailak University, Tha Sala, Thailand; 2 Center of Excellence in Tropical Pathobiology, Walailak University, Tha Sala, Thailand; 3 Department of Microbiology and Immunology, Faculty of Tropical Medicine, Mahidol University, Bangkok, Thailand; 4 Siriraj Center of Research Excellence in Allergy and Immunology, Faculty of Medicine Siriraj Hospital, Mahidol University, Bangkok, Thailand; 5 Center of Excellence on Therapeutic Proteins and Antibody Engineering, Department of Parasitology, Faculty of Medicine Siriraj Hospital, Mahidol University, Bangkok, Thailand; 6 Biomedical Research Incubator Division, Department of Research, Faculty of Medicine Siriraj Hospital, Mahidol University, Bangkok, Thailand; 7 Biodesign Innovation Program, Department of Parasitology, Faculty of Medicine Siriraj Hospital, Mahidol University, Bangkok, Thailand; Islamic Azad University, IRANISLAMIC REPUBLIC OF

## Abstract

The rise of multidrug-resistant pathogens such as *Staphylococcus aureus* and *Pseudomonas aeruginosa* has driven the search for novel antimicrobial agents with enhanced efficacy and reduced toxicity. Tachyplesin I (TP-I), a β-sheet antimicrobial peptide isolated from horseshoe crab hemocytes, is known for its broad-spectrum activity but is limited by the presence of cysteine-rich disulfide bonds. In this study, we evaluated two synthetic analogs: CDT (Cysteine-Deleted Tachyplesin I) and CRDT (Cysteine- and Arginine-Deleted Tachyplesin Analog), designed to simplify the structure and reduce production cost while maintaining or enhancing bioactivity. The antimicrobial efficacy of CDT and CRDT was assessed against *S. aureus* and *P. aeruginosa* through minimum inhibitory concentration (MIC) and minimum bactericidal concentration (MBC) assays. CRDT demonstrated potent antimicrobial activity, with enhanced membrane-disruptive effects visualized via scanning electron microscopy (SEM), especially in *P. aeruginosa*. Molecular docking revealed strong binding affinities between CRDT and key QS regulators—SarA in *S. aureus*, and LasR in *P. aeruginosa*—supporting its ability to interfere with bacterial communication systems, while qRT-PCR analysis showed significant downregulation of QS-related genes (*agrA*, *sarA*, *hla*, *algD* and *pelA*). These findings suggest that CRDT not only exhibits direct bactericidal activity but also interferes with QS-mediated communication, making it a promising candidate for the development of dual-action antimicrobial therapeutics targeting both bacterial viability and virulence.

## Introduction

Antibiotic resistance has become one of the most pressing challenges in global healthcare, with pathogens such as *Staphylococcus aureus* and *Pseudomonas aeruginosa* leading the charge in hospital-acquired infections. These opportunistic bacteria possess remarkable survival strategies—including the ability to form biofilms and regulate virulence through quorum sensing (QS) that make them highly persistent and difficult to eradicate [1–4]. Targeting QS pathways is a promising anti-virulence strategy. Here we focus on the key nodes: in *S. aureus*, the agr system and the global regulator SarA coordinate RNAIII-dependent toxins such as hla; in *P. aeruginosa*, the Las/Rhl hierarchy (with Pqs cross-talk) controls biofilm and virulence outputs including algD and pelA. These loci are attractive because modulating them can attenuate pathogenicity without directly selecting for resistance. A one-page overview of these circuits and the hypothesized CRDT interference points is provided in S1 Fig [5–7].

Among promising therapeutic approaches, antimicrobial peptides (AMPs) offer significant advantages due to their rapid membrane-disrupting activity, broad-spectrum efficacy, and low resistance potential. One such peptide, Tachyplesin I (TP-I), is a 17-residue β-hairpin AMP originally isolated from the hemocytes of the horseshoe crab (*Tachypleus tridentatus*) [8]. The amino acid sequence, KWCFRVCYRGICYRRCR-NH₂, confers potent antimicrobial activity against both Gram-positive and Gram-negative bacteria, primarily attributed to its amphipathic structure stabilized by two intramolecular disulfide bonds. However, its clinical potential is hindered by its hemolytic toxicity, which arises from its strong cationic character and membrane interaction [9,10]. To reduce toxicity while retaining function, we evaluated two rationally simplified analogs: a Cysteine-Deleted Tachyplesin I (CDT) and a derivative lacking Arg14–15 (CRDT), designed to moderate charge clustering and membrane interactions that may contribute to hemolysis while preserving antibacterial and potential anti-QS properties [11–15].

This study assesses antibacterial activity of CDT and CRDT against *S. aureus* ATCC 25923 and *P. aeruginosa* PAO1 by MIC/MBC testing; peptide-induced morphological changes by scanning electron microscopy (SEM); sub-MIC effects of CRDT on QS-associated transcripts—*sarA*, *agrA*, *hla* in *S. aureus* and *algD*, *pelA* in *P. aeruginosa*—by qRT-PCR; and plausible target engagement by in silico docking of CRDT to SarA and LasR. Together, these experiments were designed to position CRDT as a dual-action therapeutic agent capable of disrupting both bacterial survival and virulence, with improved safety and clinical relevance.

## Materials and methods

### Ethical approval

The study was approved by the Ethics Committee in Human Research, Faculty of Tropical Medicine, Mahidol University (Protocol No. MUTM 2023-082-01; approval date 07/12/2023). Five healthy adults were recruited 01/05/2024 and 31/08/2024; each provided a single 20-mL donation solely for the erythrocyte hemolysis assay. Written informed consent was obtained and documented for all participants.

Whole blood was collected into heparinized tubes, processed within 2 h, and erythrocytes were washed in PBS, resuspended, and stored at 4°C. RBC suspensions were used within 24 h; no other human samples or data were collected. All procedures complied with the Declaration of Helsinki and institutional guidelines.

### Bacterial strains and growth conditions

*Staphylococcus aureus* ATCC 25923 and *Pseudomonas aeruginosa* PAO1 were used as representative Gram-positive and Gram-negative strains, respectively. Both strains were cultured in Mueller-Hinton Broth (MHB) at 37°C with shaking (200 rpm) overnight before being adjusted to the desired inoculum density (~1 × 10⁶ CFU/mL) for susceptibility testing [16].

### Preparation of antimicrobial peptides

The native cysteine-deleted Tachyplesin I (CDT) and its cysteine- and arginine-deleted analog (CRDT) were commercially synthesized by GenScript (Nanjing, China) at >95% purity. The amino acid sequence of CDT is KWCFRVCYRGICYRRCR, while the modified CRDT sequence is KWYGYGYGIGYRYR. Lyophilized peptides were reconstituted in sterile distilled water to obtain stock solutions, which were then filter-sterilized using 0.22 μm syringe filters and stored at –20°C until use. Working concentrations were freshly prepared prior to each experiment by performing serial two-fold dilutions in MHB.

### Minimum inhibitory concentration (MIC) assay and Minimum bactericidal concentration (MBC) assay

The antimicrobial activity of CDT and CRDT was evaluated by determining their minimum inhibitory concentration (MIC) and minimum bactericidal concentration (MBC), based on the broth microdilution method with slight modifications as previously described in the literature [17], with minor modifications. Briefly, 96-well microtiter plates were prepared with two-fold serial dilutions of CDT and CRDT (ranging from 0.15 to 2.5 mg/mL). An equal volume of bacterial suspension (~1 × 10⁶ CFU/mL) was added to each well, and plates were incubated at 37°C for 24 hours. The MIC was defined as the lowest peptide concentration at which no visible bacterial growth was observed. To determine the MBC, 10 µL aliquots from wells showing no visible growth in the MIC assay were plated on Mueller-Hinton agar and incubated at 37°C for 24 hours. The MBC was defined as the lowest concentration of CDT or CRDT that resulted in ≥99.9% reduction in viable bacterial count compared to the initial inoculum, as evidenced by the absence of colony formation on the agar plates.

### Scanning electron microscopy for morphological analysis

To investigate the effect of peptides CDT or CRDT on bacterial morphological changes, *Staphylococcus aureus* ATCC 25923 and *Pseudomonas aeruginosa* PAO1 treated with each peptide were subjected to scanning electron microscopy (SEM) analysis [18]. Overnight cultures were grown in MHB at 37°C and adjusted to a final density of approximately 1 × 10⁶ CFU/mL. Bacterial suspensions were then treated with either CDT or CRDT at concentrations corresponding to their MIC and MBC values. Untreated bacterial suspensions served as negative controls. After 24 hours of incubation at 37°C, treated and control cells were harvested by centrifugation and washed twice with phosphate-buffered saline (PBS, pH 7.4). The cell pellets were fixed with 2.5% glutaraldehyde at 4°C overnight. Following fixation, samples were washed with PBS and sequentially dehydrated in a graded ethanol series (30%, 50%, 70%, 90%, and 100%), with each step lasting 10 minutes. The dehydrated cells were mounted onto sterile coverslips affixed to SEM stubs, air-dried, and sputter-coated with a thin layer of gold under vacuum. Samples were examined using a field emission scanning electron microscope (FE-SEM) operated at an accelerating voltage of 2.00 kV and a working distance of approximately 8.5 mm. Images were captured at magnifications of 5,000× and 10,000× to assess changes in bacterial cell morphology and surface integrity following peptide treatment.

### Quantitative reverse transcription PCR for quorum sensing gene expression

To investigate the effects of peptide treatment on quorum sensing (QS) regulation in *Staphylococcus aureus* and *Pseudomonas aeruginosa*, the expression of QS-associated genes was analyzed using quantitative reverse transcription PCR (qRT-PCR). In *S. aureus*, the target genes included *agrA*, *sarA*, and *hla*, while in *P. aeruginosa*, *algD* and *pelA* were evaluated. Bacterial cultures were grown in MHB supplemented with sub-inhibitory concentrations (½ × , ¼ × , and ⅛ × MIC) of CDT or CRDT peptides at 37°C for 16 hours. Following incubation, cells were harvested by centrifugation. Total RNA was extracted using the Presto™ Mini RNA Bacteria Kit (Geneaid, Taiwan), following the manufacturer protocol. Briefly, cell pellets were resuspended in Bacteria Lysis Buffer, treated with lysozyme (2 mg/mL), and further lysed by adding RB Buffer and β-mercaptoethanol. After brief incubation, lysates were centrifuged, and the supernatants were mixed with 70% ethanol and loaded onto RB spin columns. Following the wash steps, RNA was eluted in 50 μL of RNase-free water. RNA concentration and purity were evaluated using a NanoDrop spectrophotometer, and RNA samples were stored at –80°C until use.

For cDNA synthesis and amplification, qRT-PCR was performed in a 22 μL total reaction volume containing 10 μL of 2 × KAPA SYBR^®^ FAST qPCR Master Mix, 2 μL of gene-specific primer mix (forward and reverse primers at a final concentration of 100–400 nM), 4 μL of 20 × diluted cDNA, 2 μL of RNA template (0.01–1 μg), and 4 μL of nuclease-free water. The primer sequences used in this study are listed in Table 1. Cycling conditions followed kit recommendations with a melt-curve to confirm single products. Each sample was run in technical triplicate from ≥3 biological replicates. Ct values were normalized to 16S rRNA and expressed relative to the untreated control using the 2^^–ΔΔCt^ method. Primer efficiencies (90–110%) and single-peak melt curves met acceptance criteria; outlier replicates were excluded only for clear technical failure. Statistical testing is described in the Data Analysis section [23].

**Table 1:** Primer sequences used for qRT-PCR analysis.

Target Gene	Organism	Forward primer (5’ → 3’)	Reverse primer (5’ → 3’)	Reference
*agrA*	*S. aureus*	ACGTGGCAGTAATTCAGTGTATGTT	GGCAATGAGTCTGTGAGATTTTGT	[19]
*sarA*	*S. aureus*	GCTGTATTGACATACATCAGCGAA	CGTTGTTTGCTTCAGTGATTCGT	[19]
*hla*	*S. aureus*	ACAATTTTAGAGAGCCCAACTGAT	TCCCCAATTTTGATTCACCAT	[20]
*pelA*	*P. aeruginosa*	CCTTCAGCCATCCGTTCTTCT	TCGCGTACGAAGTCGACCTT	[21]
*algD*	*P. aeruginosa*	GAGGAATACCAGCTGATCCGG	CACCGAGTTCAAGGACCTGAA	[22]
*16S rRNA*	*S. aureus*	TGATCCTGGCTCAGGATGA	TTCGCTCGACTTGCATGTA	[19]
*16S rRNA*	*P. aeruginosa*	CAAAACTACTGAGCTAGAGTACG	TAAGATCTCAAGGATCCCAACGGCT	[21]

### Computerized simulation for predicting interaction interfaces of QS receptors with CRDT

To elucidate the molecular mechanism by which the CRDT peptide may interfere with quorum sensing (QS) in *Pseudomonas aeruginosa* and *Staphylococcus aureus*, an in silico docking approach was employed to predict the contact residues and interface regions involved in its interaction with the QS receptors LasR (*P. aeruginosa,* PDB 2UV0) and SarA (*S. aureus*, PDB 2FNP).

**Receptor/ligand preparation.** Crystal structures were cleaned (cofactors/ligands/waters removed), hydrogens added, and Gasteiger charges assigned in AutoDockTools v1.5.6; protonation was set to pH 7.4. The CRDT peptide was built from sequence, converted with Open Babel v3.x, and energy-minimized; rotatable side chains were kept flexible while the backbone was restrained to limit the search space. All files were exported as.pdbqt [24].

**Search space and settings.** Grid boxes were centered on the LasR ligand-binding domain and on the SarA DNA-binding surface (α3–α4 interface), each sized to encompass the pocket plus ~10 Å padding in X/Y/Z. Exhaustiveness = 8 (default); 10 poses/receptor were generated. Docking simulations were performed using AutoDock Vina implemented within the PyRx 0.8 platform [25–27]. The docking grid was defined to cover the known ligand-binding region of each receptor, ensuring adequate space for peptide flexibility.

**Pose selection and analysis.** Poses were ranked by Vina affinity and cluster density (RMSD ≤ 2.0 Å). The top biologically plausible pose—consistent with known binding regions—was selected for reporting. Interactions (H-bonds, electrostatics, hydrophobics, π-contacts) were inspected in Discovery Studio Visualizer v3.5; contacting residues within 5 Å were annotated and mapped to functional domains. Software versions and parameter files are listed in Methods and deposited with the minimal dataset [28]. These residues were subsequently mapped to functional domains of LasR and SarA to assess potential biological relevance. Structural mapping revealed that CRDT interacted predominantly with the β-turn and α-helix regions surrounding the autoinducer-binding pocket in LasR, while in SarA, key contacts were found within the DNA-binding domain, particularly helices α3 and α4. Residues involved in multiple interaction modes were classified as core interface residues, suggesting their functional importance in CRDT-mediated QS inhibition.

#### Hemolytic activity assay for biocompatibility assessment of CRDT.

To assess the cytotoxicity and biocompatibility of CRDT for potential biomedical applications, a hemolysis assay was conducted using five independent samples of healthy human blood. Residual whole blood samples were centrifuged, and the RBCs were collected, washed twice with isotonic washing buffer, and centrifuged at 1,000–2,000 rpm for 5 minutes. After the final wash, the erythrocyte pellet was resuspended in dilution buffer to prepare a 5% RBC suspension.

For the hemolysis assay, 100 μL of the RBC suspension was mixed with 100 μL of peptide solution (CRDT) at various concentrations. The phosphate-buffered saline (PBS; 0.15 M NaCl, 0.05 M phosphate buffer, pH 7.4) was used as the negative control (0% hemolysis), and 0.1% Triton X-100 served as the positive control (100% hemolysis). Samples were incubated at 37°C for 1 hour. After incubation, samples were centrifuged to pellet intact cells. The supernatants were transferred to a new 96-well plate, and hemoglobin release—indicative of RBC lysis—was quantified by measuring absorbance at 415 nm with 450 nm as the reference wavelength, following the method described by Shi et al. (2018) [29]. The percentage of hemolysis was calculated relative to the controls using the standard formula. This assay enabled evaluation of the membrane-disruptive potential of the peptides on human erythrocytes, serving as an important indicator of their biocompatibility.

### Statistical analysis

All data are expressed as the mean ± standard deviation (SD) from three independent experiments. Differences between treatment groups and the untreated control were assessed using one-way analysis of variance (ANOVA), followed by Tukey’s multiple comparison test. Statistical analysis was performed using GraphPad Prism version 9 (GraphPad Software, La Jolla, CA, USA). A *p*-value less than 0.05 was considered indicative of a statistically significant difference.

## Results

### Antimicrobial activity of CDT and CRDT against *Staphylococcus aureus* and *Pseudomonas aeruginosa*

The minimum inhibitory concentration (MIC) and minimum bactericidal concentration (MBC) assays demonstrated that *Staphylococcus aureus* ATCC 25923 was more susceptible to both native CDT and CRDT compared to *Pseudomonas aeruginosa* PAO1. The MIC values for *S. aureus* were 0.625 mg/mL for CDT and 1.25 mg/mL for CRDT, while the corresponding MBC values were 1 mg/mL and 1.25 mg/mL, respectively. In contrast, *P. aeruginosa* PAO1 exhibited reduced susceptibility, with both MIC and MBC values exceeding 1 mg/mL for CDT and 1.25 mg/mL for CRDT. These results suggest that while both peptides exhibit antibacterial activity, *S. aureus* is more sensitive to their effects than *P. aeruginosa*, and that CRDT retains bactericidal activity comparable to native CDT, particularly against Gram-positive bacteria. To contextualize potency, we note that reference AMPs such as melittin typically inhibit *S. aureus* at low–tens of μg/mL but are strongly hemolytic, and polymyxin B/colistin act against *P. aeruginosa* in the low-μg/mL range. By comparison, CRDT requires mg/mL concentrations but shows minimal hemolysis, supporting its positioning as a low-toxicity anti-virulence/adjunct candidate rather than a primary bactericidal agent [30,31].

The higher MIC/MBC in *P. aeruginosa* are consistent with known AMP-evasion mechanisms, including LPS remodeling via the Arn/L-Ara4N pathway, efflux, proteolytic degradation, and induction of AMP-responsive two-component systems (e.g., ParRS/CprRS) that remodel the envelope and raise the barrier to membrane disruption [32].

### Morphological alteration by CRDT against *Staphylococcus aureus* and *Pseudomonas aeruginosa*

Scanning electron microscopy (SEM) revealed distinct morphological changes in both *S. aureus* ATTCC 25923 and *P. aeruginosa* PAO1 after treatment with CRDT (primary focus) and, briefly for comparison, CDT. SEM was used as a qualitative readout of envelope damage. Images show representative fields from three biological replicates; magnifications and scale bars are indicated.

#### *S. aureus* morphology.

**Fig 1:**
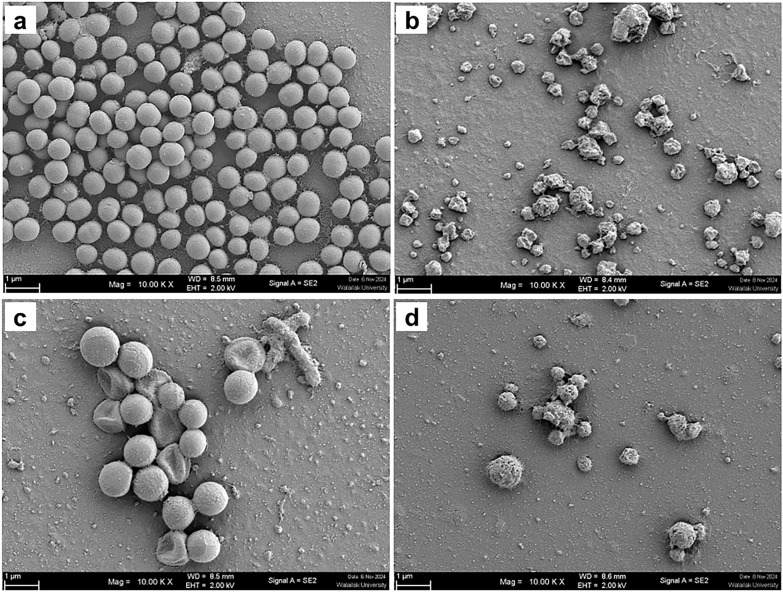
Scanning electron micrographs of *Staphylococcus aureus* ATCC 25923 treated with CDT and CRDT. **(a)** Untreated control showing intact, smooth coccal morphology. **(b)** CRDT-treated cells (1.25 mg/mL) exhibiting extensive disruption, including membrane rupture and cell collapse. **(c)** Cells treated with native CDT (0.625 mg/mL) showing mild surface irregularities. **(d)** Cells treated with native CDT (1.0 mg/mL) showing increased surface damage and partial lysis.

Untreated *S. aureus* ATCC 25923 exhibited typical smooth, spherical coccal shapes densely packed in clusters (Fig 1a). CRDT (1.25 mg/mL) produced pronounced membrane disruption, including surface rupture, collapse, and loss of cellular integrity (Fig 1b). By contrast, CDT caused milder changes: surface roughening at 0.625 mg/mL and partial collapse/blebbing at 1.0 mg/mL (Fig 1c–1d).

#### *P. aeruginosa* morphology.

**Fig 2:**
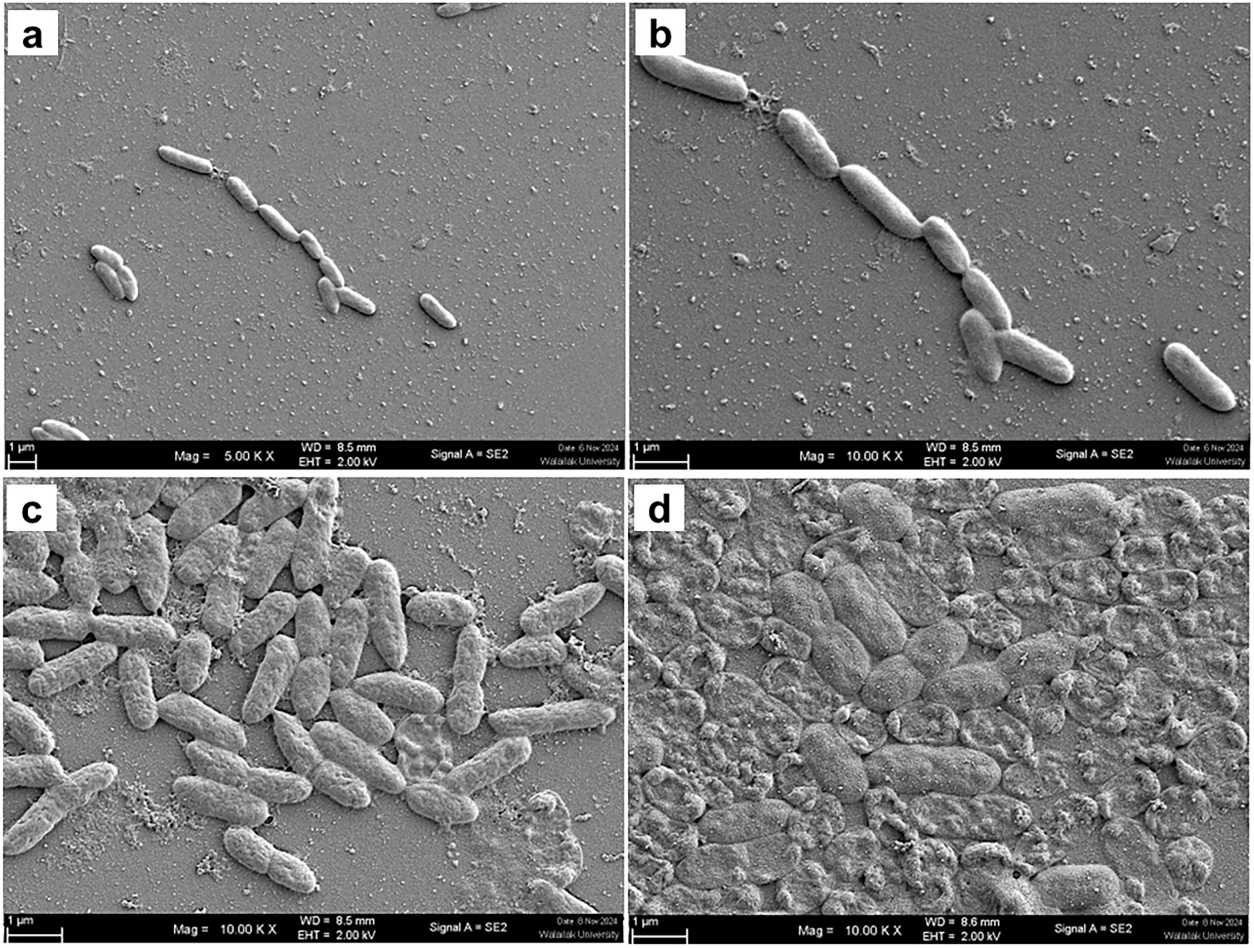
Scanning electron micrographs of *Pseudomonas aeruginosa* PAO1 treated with CDT and CRDT (qualitative readout). Panels show representative fields from three biological replicates; magnifications and scale bars are indicated. **(a–b)** Untreated control cells with intact, smooth rod morphology. **(c)** CDT (comparator)–treated cells showing moderate aggregation and surface distortion with overall envelope largely preserved. **(d)** CRDT (1.25 mg/mL) producing granular/pitted envelopes and membrane collapse, consistent with severe envelope damage.

Untreated *P. aeruginosa* PAO1 showed intact, elongated rods with smooth surfaces (Fig 2a–2b). CRDT induced granular/pitted envelopes and membrane collapse, consistent with severe stress and architectural failure (Fig 2d). CDT mainly yielded cell aggregation and superficial distortion, with overall structure largely preserved (Fig 2c).

Across multiple fields of view and replicates, CRDT consistently produced more extensive envelope damage than CDT in both species, supporting a membrane-targeting mechanism and aligning with its greater antibacterial effects.

### Effect of CDT and CRDT Peptides on Quorum Sensing Gene Expression in *Staphylococcus aureus* and *Pseudomonas aeruginosa*

Quantitative real-time PCR (qRT-PCR) revealed that treatment with CRDT at sub-MIC concentrations (½ × , ¼ × , and ⅛ × MIC) significantly downregulated the expression of key QS-related genes in both *Staphylococcus aureus* and *Pseudomonas aeruginosa*.

**Fig 3:**
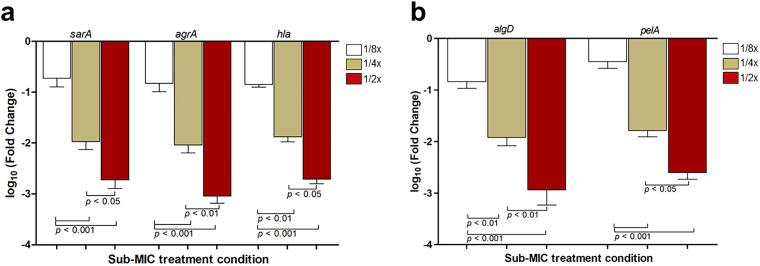
Effect of CRDT on the expression of quorum sensing-related genes in *Staphylococcus aureus* and *Pseudomonas aeruginosa.* Quantitative real-time PCR (qRT-PCR) analysis of log₁₀-transformed fold changes in gene expression after treatment with sub-MIC concentrations (½ × , ¼ × , and ⅛ × MIC) of CRDT. **(a)** Expression levels of *sarA*, *agrA*, and *hla* in *S. aureus* ATCC 25923. **(b)** Expression levels of *algD* and *pelA* in *P. aeruginosa* PAO1. Data represent the mean ± standard deviation (SD) of three independent experiments. Statistical significance was assessed using one-way ANOVA followed by Tukey’s post hoc test. Differences were considered significant at *p* < 0.05 (*), *p* < 0.01 (**), and *p* < 0.001 (***).

In *S. aureus*, the genes *sarA, agrA,* and *hla* exhibited a dose-dependent reduction in expression, with the strongest suppression observed at ½ × MIC. Log₁₀-transformed fold change values showed that *sarA* and *agrA* expression dropped by more than 2 logs at the highest dose (*p* < 0.001), while *hla* also showed significant repression (**p* *< 0.05 to *p* < 0.001) (Fig 3a). Similarly, in *P. aeruginosa*, the QS regulatory genes *algD* and *pelA* were markedly downregulated by CRDT treatment. Notably, both genes showed more than 2-log reduction at ½ × MIC, with statistically significant decreases observed at all sub-MIC levels (**p* *< 0.01 to *p* < 0.001) (Fig 3b). These results indicate that CRDT interferes with the quorum sensing pathways of both Gram-positive and Gram-negative pathogens in a dose-dependent manner, highlighting its potential as an effective anti-virulence agent targeting QS-regulated gene expression.

### Molecular docking of CRDT with QS regulators in *Pseudomonas aeruginosa* and *Staphylococcus aureus*

Molecular docking simulations revealed that the CRDT peptide exhibits strong binding affinities and forms stable complexes with key QS regulators from both Gram-negative *Pseudomonas aeruginosa* (LasR) and Gram-positive *Staphylococcus aureus* (SarA). These interactions highlight the dual-targeting potential of CRDT as a quorum sensing inhibitor capable of modulating virulence and biofilm formation pathways in both organisms. The predicted binding energy of CRDT was −8.4 kcal/mol with LasR and −7.9 kcal/mol with SarA, suggesting energetically favorable interactions in both systems. In both complexes, hydrogen bonding was the dominant interaction type, supported by electrostatic forces and hydrophobic contacts, which collectively contribute to ligand stability and specificity (Tables 2 and 3).

**Table 2:** Predicted intermolecular interactions between CRDT and SarA quorum sensing receptor in *Staphylococcus aureus.*

Target protein	Interacting residues		Type of interaction	Distance (Å)	Biological implication
	Receptor	CRDT ligand			
SarA (*S. aureus*)	SER99	ARG7	Hydrogen bonding	2.37	Stabilizes ligand entry through polar contact in the active region of SarA.
	ILE103	ILE9	Hydrogen bonding	2.55, 3.10	Reinforces ligand anchoring via backbone–backbone interaction.
	TYR118	TYR10	Hydrogen bonding	3.03	Contributes to peptide orientation through polar aromatic interaction.
	ASN146	VAL5	Hydrogen bonding	2.87	Promotes positioning of hydrophobic segment through polar stabilization.
	GLU148	LYS1	Electrostatic	4.37	Forms attractive ionic interaction, enhancing peptide affinity.
			Hydrogen bonding	3.68	Supports electrostatic engagement through complementary polar contact.
	GLU148	PHE3	Hydrogen bonding	3.25	Contributes to ligand stabilization near the regulatory cleft.
	GLU150	LYS1	Hydrogen bonding	2.19, 2.80	Promotes strong hydrogen bonding, potentially affecting SarA conformation.
	ASP155	LYS1	Electrostatic	4.82	Forms long-range ionic interaction that may influence ligand binding energy.
	ASN161	ARG13	Hydrogen bonding	2.22	Stabilizes the N-terminal side of the peptide through tight polar contact.
	ILE222	ARG13	Hydrogen bonding	2.35, 2.57	Anchors central peptide residues near the DNA-binding region.
	GLU223	ARG13	Hydrogen bonding	1.88, 2.02	Enables strong interaction near the recognition loop of SarA.
	LEU224	ILE9	Hydrogen bonding	2.18	Enhances local stabilization of hydrophobic residues through polar support.

**Table 3:** Predicted intermolecular interactions between CRDT and LasR quorum sensing receptor in *Pseudomonas aeruginosa.*

Target protein	Interacting residues		Type of interaction	Distance (Å)	Biological implication
	Receptor	CRDT ligand			
LasR (*P. aeruginosa*)	LEU10	ARG13	Hydrogen bonding	2.59	Enhances anchoring in the lower region of the binding pocket.
	GLU11	ARG13	Hydrogen bonding	2.98	Additional short-range interaction for ligand stabilization.
	SER13	ARG13	Hydrogen bonding	2.75, 3.77	Forms directional polar interaction aiding in ligand docking and directional polar interaction contributing to surface affinity.
	LYS16	TRP2	Hydrogen bonding	1.89	Strong polar contact with aromatic sidechain, possibly stabilizing aromatic stacking.
	LYS16	ARG7	Hydrogen bonding	2.28	Helps stabilize ligand entry via charged–polar contact.
	PRO41	TYR10	Hydrogen bonding	3.59	Provides hydrophobic and polar alignment near a flexible loop.
	ASN49	ARG7	Hydrogen bonding	2.49	Stabilizes local ligand orientation near the receptor interface.
	ASN49	TYR10	Hydrogen bonding	2.69	Supports proper orientation of aromatic residue in the binding groove.
	ALA50	ARG7	Hydrogen bonding	2.23	Anchors the ligand through backbone-sidechain H-bonding at the surface.
	GLY54	LYS1	Hydrogen bonding	3.31	Stabilizes polar head via flexible glycine–lysine contact.
			Hydrogen bonding	2.58, 2.70	Close polar contact enhancing ligand affinity.
	ASN55	LYS1	Hydrogen bonding	3.02	Assists hydrogen network formation for ligand anchoring.
	TYR56	ARG4	Hydrogen bonding	2.17	Critical for positioning positively charged ligand sidechains.
	TYR56	LYS1	Hydrogen bonding	2.32, 2.71	Contributes to dynamic binding through polar interactions and maintains positioning of polar moiety within the pocket.
	ASP65	VAL5	Hydrogen bonding	3.28	Helps orient hydrophobic core near polar zone of the receptor.
	ALA166	TYR10	Hydrogen bonding	3.10	Supports edge-of-pocket orientation of ligand aromatic ring.

**Fig 4:**
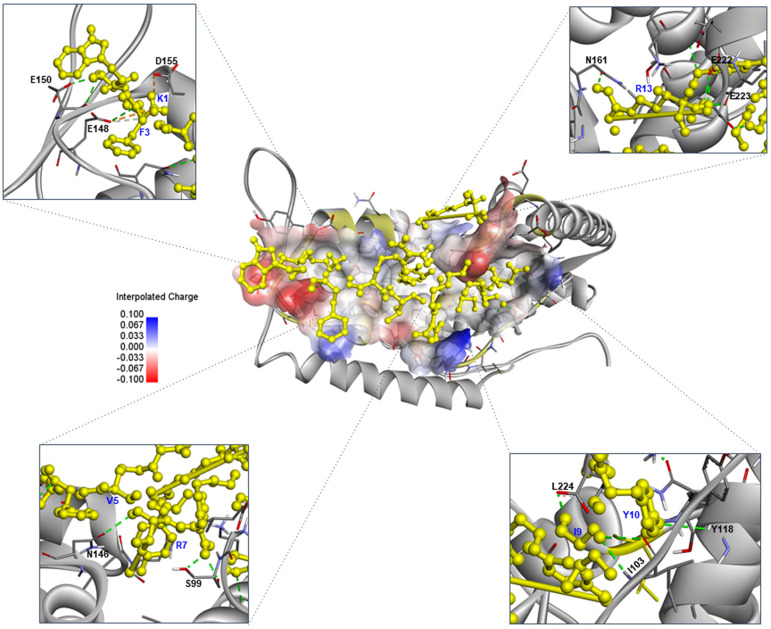
Computerized interaction of the CRDT peptide and the SarA quorum sensing regulator of *Staphylococcus aureus*, and residues predicted to form contact interfaces. The central panel displays the docking of the CRDT peptide (yellow stick representation) onto the SarA protein (grey ribbon), with electrostatic surface potential visualized (red indicates negatively charged regions, blue indicates positively charged regions). The inset panels illustrate specific binding interfaces.

**Fig 5:**
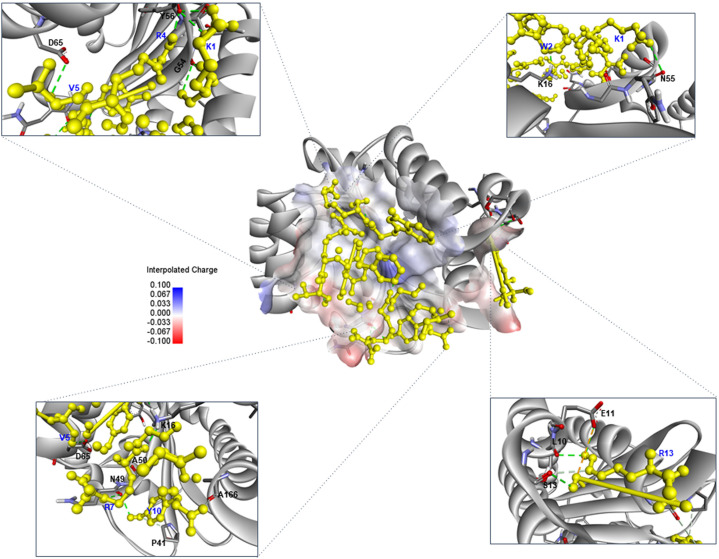
Computerized interaction of the CRDT peptide and the LasR quorum sensing regulator of *Pseudomonas aeruginosa*, and residues predicted to form contact interfaces. The docking of CRDT (yellow stick model) into the LasR receptor (grey ribbon) is shown in the center, with electrostatic surface mapping illustrating charged receptor regions. Insets provide detailed visualizations of peptide–receptor contacts.

In the SarA–CRDT complex, CRDT targeted residues within the DNA-binding interface of SarA, including SER99, ILE103, TYR118, ASN146, GLU148, and GLU150. Multiple hydrogen bonds were formed between CRDT residues ARG7, ILE9, VAL5, and LYS1, anchoring the peptide to helices α3 and α4, which are critical for SarA transcriptional activity. Electrostatic interactions, such as those between GLU148 and LYS1, and between ASP155 and LYS1, further stabilized the peptide in regions essential for DNA-binding. These contacts suggest that CRDT may inhibit SarA-mediated gene regulation by directly occupying its functional domain. In the LasR–CRDT complex, CRDT was accommodated within the canonical ligand-binding domain of LasR, forming key hydrogen bonds with residues such as GLU11, SER13, ASN49, TYR56, and ALA50. Notably, ARG13 and TYR10 from the peptide contributed to directional polar interactions, while LYS1 and TRP2 engaged in close contacts with GLY54 and LYS16, enhancing peptide anchoring and orientation. These residues cluster around the β-turn and α-helix regions known to form the natural autoinducer-binding cleft, indicating that CRDT may interfere with native ligand recognition and quorum signal propagation in *P. aeruginosa*. Additionally, residues PRO41 and ALA166 contributed to edge-of-pocket positioning of the peptide, supporting both hydrophobic and polar contact stabilization. Altogether, these findings suggest that CRDT is capable of forming a stable and specific complex with the LasR receptor and may act as a competitive quorum sensing inhibitor by occupying the autoinducer-binding cleft and blocking native ligand recognition. The docking visualizations (Figs 4 and 5) highlight the structural positioning of CRDT across the receptor surfaces of both SarA and LasR, with favorable electrostatic complementarity and extensive interaction networks in each case. Together, these findings demonstrate that CRDT can interact specifically and stably with QS regulators in both pathogens, offering a promising strategy for dual-species quorum sensing interference and potential antimicrobial development.

### Hemolytic activity of CDT and CRDT on human red blood cells

The hemolytic potential of CRDT was assessed to evaluate their safety toward mammalian cells. Human red blood cells (RBCs) were incubated with increasing concentrations of peptide, and hemolysis was quantified relative to 0.1% Triton X-100, used as a positive control for 100% lysis. CRDT showed negligible hemolytic activity (<1%) at all tested concentrations ranging from 0.15 to 2.5 mg/mL, indicating a favorable safety profile toward erythrocytes. Hemolysis levels remained below 5% for both peptides, with no statistically significant difference compared to the negative control (PBS) or between the two peptides (*p* > 0.05). The complete lysis observed in the positive control confirmed the validity of the assay. These findings suggest that CRDT exhibits minimal cytotoxicity toward human RBCs, supporting their potential for therapeutic applications.

## 4. Discussion

Quorum sensing (QS) underpins virulence, biofilm maturation, and host interaction in *Staphylococcus aureus* and *Pseudomonas aeruginosa*; targeting QS offers an anti-virulence route that can complement antibiotics [1,33,34]. We evaluated a cysteine- and arginine-deleted Tachyplesin analog (CRDT) for antibacterial activity and QS interference. Across assays, CRDT inhibited growth—more strongly in *S. aureus* than *P. aeruginosa*—and suppressed QS-associated transcripts in a dose-dependent fashion, supporting its potential as a low-toxicity anti-virulence lead.

### Susceptibility and membrane effects

*S. aureus* was more susceptible than *P. aeruginosa*, consistent with AMP behavior in Gram-positives lacking an outer membrane [14,15,35]. In contrast, *P. aeruginosa* outer membrane, LPS remodeling, efflux, and proteases likely contribute to the higher MIC/MBCs. SEM (qualitative) corroborated a membrane-targeting effect, with CRDT producing more extensive envelope disruption than CDT in both species. Thus, removing cysteine and Arg14–15 preserved bactericidal activity against *S. aureus* without increasing hemolysis. To enhance Gram-negative activity, we outline pragmatic next steps: (i) sequence tuning—moderate increases in hydrophobic moment, N-terminal acylation, Orn/Dab substitutions, and limited D-residue/cyclization to improve LPS interaction and protease resistance; (ii) formulation/delivery—liposomal or cationic-nanoparticle encapsulation and co-administration with outer-membrane permeabilizers or efflux inhibitors; and (iii) combination therapy—pairing CRDT with Gram-negative antibiotics to leverage OM perturbation while retaining anti-QS engagement.

### Mechanistic link between docking and gene suppression

Docking places CRDT in functionally relevant regions of both SarA and LasR. In *S. aureus*, CRDT bound predominantly within the DNA-binding region of SarA, with key interactions involving GLU148, GLU150, ASP155, ASN161, ILE222, and GLU223. These residues are located within helices α3 and α4, which form the DNA recognition interface of SarA [24–36]. The peptide formed hydrogen bonds with multiple residues including SER99, ILE103, TYR118, and ASN146, suggesting broad surface interaction and strong anchoring. In *P. aeruginosa*, CRDT formed hydrogen bonds with ARG13, ASN49, ALA50, GLY54, and TYR56, localizing around the β-turn and α-helix regions that constitute the autoinducer-binding domain of LasR [7,37]. These regions are known to be critical for ligand recognition and receptor activation. Residues such as TYR10 and LYS16 further contributed to aromatic stacking and anchoring within the binding cleft, enhancing the stability and specificity of the CRDT–LasR complex through π–π and electrostatic interactions [38–41]. These interactions suggest that CRDT occupies the canonical autoinducer pocket and may competitively inhibit LasR signaling, thereby disrupting QS-mediated gene regulation and virulence expression. Notably, several residues, such as ARG13 and LYS1, were involved in multiple binding interactions in both organisms, and were therefore identified as core interface residues, indicating their central role in CRDT binding and potential QS inhibition. Structural mapping of these interactions revealed that CRDT does not bind randomly across the receptor surfaces, but instead targets functionally conserved domains in both QS proteins. This domain-specific binding highlights CRDT potential as a rationally designed anti-QS peptide. The occupation of the LasR autoinducer pocket and the SarA DNA-binding domain by CRDT is highly indicative of its role in transcriptional interference—corroborated by qRT-PCR findings that showed significant downregulation of QS-associated genes such as *lasI*, *lasR*, *rhlI*, *rhlR, agrA*, *sarA*, and *hla*. However, docking is predictive, not proof of binding, and qRT-PCR reflects downstream pathway attenuation. To validate mechanism, we will (i) quantify Las/Rhl activity using lasB-gfp and rhlA-gfp reporters, (ii) test SarA–promoter disruption by EMSA, and (iii) measure CRDT–LasR/SarA binding by SPR/ITC.

### Context of anti-QS activities

The observed reductions in *sarA*, *agrA*, and *hla* expression in *S. aureus* indicate that CRDT interferes with both global and accessory QS regulators involved in biofilm formation and virulence factor production. Notably, the dose-dependent suppression of *sarA* and *agrA*, decreases at ½ × MIC, highlights the potent anti-QS activity of CRDT in Gram-positive bacteria. This is consistent with previous findings that targeting the SarA–agr regulatory axis disrupts toxin production and pathogenic persistence [36,42].

In *P. aeruginosa*, CRDT significantly suppressed *algD* and *pelA*, key genes involved in exopolysaccharide synthesis and biofilm structural integrity. These genes are regulated by the Las and Rhl QS systems, suggesting that CRDT may indirectly or directly interfere with QS signal reception and downstream gene activation. The consistent downregulation across all tested sub-MIC concentrations supports the hypothesis that CRDT acts as a broad-spectrum QS disruptor in both Gram-positive and Gram-negative pathogens [43,44].

Importantly, CRDT exhibited minimal hemolytic activity toward human red blood cells, reinforcing its potential as a safer alternative to native CDT. When combined with its demonstrated antimicrobial efficacy and broad-spectrum membrane-disruptive effects, these findings position CRDT as a strong candidate for further development against multidrug-resistant infections. In addition, CRDT showed the ability to disrupt quorum sensing pathways in both Gram-positive and Gram-negative bacteria, likely through direct interactions with conserved functional domains of the LasR and SarA regulators.

### Context of activity and safety

Compared with reference AMPs—melittin (typical MICs against *S. aureus* in the low–tens of µg/mL, but highly hemolytic) and polymyxins/colistin (active against *P. aeruginosa* in the low-µg/mL range)—CRDT requires mg/mL exposures for direct growth inhibition yet produces negligible RBC hemolysis across 0.15–2.5 mg/mL. Taken together, this profile supports positioning CRDT primarily as an anti-virulence or adjunct agent (QS disruption with low cytotoxicity) rather than a frontline bactericidal peptide [30,31,45]. While CRDT caused negligible hemolysis across the tested range, we did not assess cytotoxicity in mammalian cell lines—a limitation of the present work. To complete the safety profile, we will perform MTT and LDH assays in representative human keratinocytes/epithelial cells and primary dermal fibroblasts with 24–48 h exposures across and above antibacterial concentrations. Until these data are available, we interpret CRDT primarily as a preliminary anti-virulence/adjunct candidate rather than a definitive bactericidal therapy.

### Resistance considerations

Potential adaptive responses include outer-membrane/LPS remodeling, efflux up-regulation, protease-mediated peptide cleavage, or mutations at regulator interaction surfaces. We note that dual action (membrane perturbation with anti-QS) could raise the evolutionary barrier, but this requires longitudinal selection experiments.

### Translational outlook

CRDT combines negligible hemolysis with predicted interference at SarA/LasR and concordant suppression of QS-regulated genes. These features support next steps in vivo (e.g., *S. aureus* skin/wound and *P. aeruginosa* lung/device-biofilm models, with combination therapy arms), pharmacokinetic/pharmacodynamic profiling across relevant routes (IV, topical, inhaled), and formulation work to enhance stability and Gram-negative activity (e.g., liposomal or polymeric nanoparticles, hydrogels, surface coatings).

### Limitations and future directions

Docking is predictive and does not prove binding. We therefore outline orthogonal validations: (i) biosensor reporters (e.g., *lasB-gfp*, *rhlA-gfp*) to confirm QS pathway inhibition; (ii) EMSA to test SarA–promoter displacement; and (iii) SPR/ITC to quantify CRDT–regulator interactions. Our SEM analyses were designed as qualitative readouts of envelope damage; we now state this explicitly and add a limitation that future work will implement pre-specified scoring, blinded field selection, and membrane-integrity assays (e.g., propidium iodide uptake, lipid vesicle leakage). In addition, we currently lack experimental structural data for CRDT. We will address this by performing circular dichroism (CD) in membrane-mimetic media (e.g., SDS/TFE micelles or small unilamellar liposomes) with temperature ramps to assess thermal stability, and—where feasible—complementary NMR or FTIR to confirm secondary structure under membrane-relevant conditions.

### Conclusion

CRDT combines modest antibacterial activity with mechanistically plausible inhibition of SarA/LasR, producing reproducible suppression of QS-regulated genes in *S. aureus* and *P. aeruginosa*. Given its low hemolysis, CRDT is best positioned as an anti-virulence/adjunct lead for preclinical evaluation, not a near-clinical therapeutic. Next steps include orthogonal binding assays (e.g., EMSA, SPR/ITC), quantitative SEM and membrane-integrity readouts, broader testing across clinical strain panels, and in-vivo efficacy and pharmacokinetic studies.

## Supporting information

S1 Fig Schematic of QS pathways in *Staphylococcus aureus* (Agr/SarA) and *Pseudomonas aeruginosa* (Las/Rhl/PQS) with proposed CRDT interference points. *P. aeruginosa* QS hierarchy. LasI synthesizes 3-oxo-C12-HSL that activates LasR, which in turn upregulates *lasB/algD* and drives Rhl and Pqs systems. RhlI produces C4-HSL for RhlR, which activates rhlA/pelA. CRDT is predicted to bind the LasR ligand pocket (red X), leading to downstream attenuation of Rhl/Pqs outputs. *S. aureus* Agr/SarA network. AIP produced by agrD/agrB activates the AgrC/AgrA two-component system, inducing RNAIII and downstream Hla. SarA regulates virulence, represses extracellular proteases, and promotes biofilm genes; it can also influence agr. CRDT is predicted to bind SarA, potentially reducing promoter occupancy and attenuating RNAIII-dependent virulence outputs.
(PNG)

S1 File Quantitative qRT-PCR datasets for *Pseudomonas aeruginosa* and *Staphylococcus aureus* under untreated and sub-MIC (½ × , ¼ × , and ⅛×) conditions. This file contains raw and processed per-replicate qRT-PCR data for *algD*, *pelA* (*P. aeruginosa*) and *sarA*, *agrA*, and *hla* (*S. aureus*) of the test compound (n = 3 replicates/condition). Columns include gene, condition, replicate, log10 fold change, ΔΔCt, and fold-change values.
(CSV)
