# Supplementary figures and images for: Antimicrobial and quorum sensing interference of a cysteine- and arginine-deleted linear Tachyplesin analog (CRDT) against Staphylococcus aureus and Pseudomonas aeruginosa
Source: PLoS One. 2025 Oct 10;20(10):e0334547. doi: 10.1371/journal.pone.0334547 (PMC12513661; doi:10.1371/journal.pone.0334547)

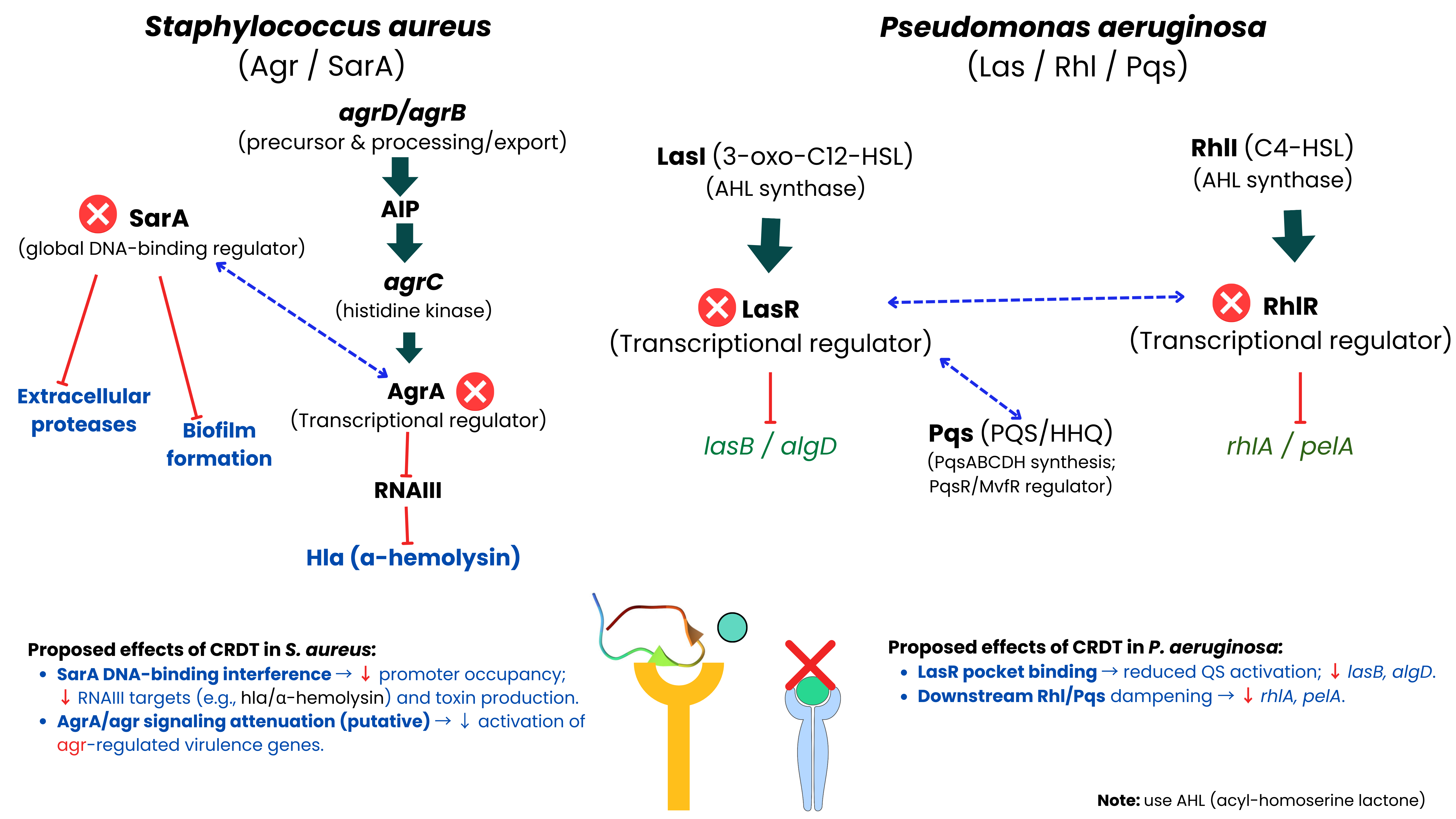

Supplement: S1 Fig — P. aeruginosa QS hierarchy. LasI synthesizes 3-oxo-C12-HSL that activates LasR, which in turn upregulates lasB/algD and drives Rhl and Pqs systems. RhlI produces C4-HSL for RhlR, which activates rhlA/pelA. CRDT is predicted to bind the LasR ligand pocket (red X), leading to downstream attenuation of Rhl/Pqs outputs. S. aureus Agr/SarA network. AIP produced by agrD/agrB activates the AgrC/AgrA two-component system, inducing RNAIII and downstream Hla. SarA regulates virulence, represses extracellular proteases, and promotes biofilm genes; it can also influence agr. CRDT is predicted to bind SarA, potentially reducing promoter occupancy and attenuating RNAIII-dependent virulence outputs. (PNG) [file pone.0334547.s001.png]
